# Pharmaceutical Industry Off-label Promotion and Self-regulation: A Document Analysis of Off-label Promotion Rulings by the United Kingdom Prescription Medicines Code of Practice Authority 2003–2012

**DOI:** 10.1371/journal.pmed.1001945

**Published:** 2016-01-26

**Authors:** Andreas Vilhelmsson, Courtney Davis, Shai Mulinari

**Affiliations:** 1 Department of Clinical Sciences, Division of Social Medicine and Global Health, Faculty of Medicine, Malmö University, Lund, Sweden; 2 Department of Gender Studies, Faculty of Social Sciences, Lund University, Lund, Sweden; 3 Department of Social Science, Health and Medicine, King’s College London, London, United Kingdom; 4 Department of Sociology, Faculty of Social Sciences, Lund University, Lund, Sweden; York University, CANADA

## Abstract

**Background:**

European Union law prohibits companies from marketing drugs off-label. In the United Kingdom*—*as in some other European countries, but unlike the United States*—*industry self-regulatory bodies are tasked with supervising compliance with marketing rules. The objectives of this study were to (1) characterize off-label promotion rulings in the UK compared to the whistleblower-initiated cases in the US and (2) shed light on the UK self-regulatory mechanism for detecting, deterring, and sanctioning off-label promotion.

**Methods and Findings:**

We conducted structured reviews of rulings by the UK self-regulatory authority, the Prescription Medicines Code of Practice Authority (PMCPA), between 2003 and 2012. There were 74 off-label promotion rulings involving 43 companies and 65 drugs. Nineteen companies were ruled in breach more than once, and ten companies were ruled in breach three or more times over the 10-y period. Drawing on a typology previously developed to analyse US whistleblower complaints, we coded and analysed the apparent strategic goals of each off-label marketing scheme and the practices consistent with those alleged goals. 50% of rulings cited efforts to expand drug use to unapproved indications, and 39% and 38% cited efforts to expand beyond approved disease entities and dosing strategies, respectively. The most frequently described promotional tactic was attempts to influence prescribers (*n* = 72, 97%), using print material (70/72, 97%), for example, advertisements (21/70, 30%). Although rulings cited prescribers as the prime target of off-label promotion, competing companies lodged the majority of complaints (prescriber: *n* = 16, 22%, versus companies: *n* = 42, 57%). Unlike US whistleblower complaints, few UK rulings described practices targeting consumers (*n* = 3, 4%), payers (*n* = 2, 3%), or company staff (*n* = 2, 3%). Eight UK rulings (11%) pertaining to six drugs described promotion of the same drug for the same off-label use as was alleged by whistleblowers in the US. However, while the UK cases typically related to only one or a few claims made in printed material, several complaints in the US alleged multifaceted and covert marketing activities. Because this study is limited to PMCPA rulings and whistleblower-initiated federal cases, it may offer a partial view of exposed off-label marketing.

**Conclusion:**

The UK self-regulatory system for exposing marketing violations relies largely on complaints from company outsiders, which may explain why most off-label promotion rulings relate to plainly visible promotional activities such as advertising. This contrasts with the US, where Department of Justice investigations and whistleblower testimony have alleged complex off-label marketing campaigns that remain concealed to company outsiders. UK authorities should consider introducing increased incentives and protections for whistleblowers combined with US-style governmental investigations and meaningful sanctions. UK prescribers should be attentive to, and increasingly report, off-label promotion.

## Abstract 2

In a document analysis of legal cases, Shai Mulinari and colleagues characterize off-label drug promotion by pharmaceutical companies in the UK.

## Editors' Summary

**Background:**

Before a pharmaceutical company can market a new prescription drug, the drug has to go through a long approval process. After extensive studies in the laboratory and in animals, the company must test the drug’s safety and efficacy in series of clinical trials in which patients with specific diseases receive the drug under carefully controlled conditions. Regulatory bodies such as the US FDA, the UK MHRA and the European Medicines Agency (EMA) then review the results of these trials and, when they are satisfied that the drug is safe and effective for the conditions in which it was tested, give the pharmaceutical company approval to market the drug in the relevant country. An important part of the approval process is the creation of the “drug label,” a detailed report that specifies the exact diseases and patient groups in which the drug can be used and the drug’s approved doses.

**Why Was This Study Done?:**

Physicians can use approved drugs “off-label.” That is, they can prescribe drugs for a different disease, in a different group of patients, or at a different dose to that specified in the label. However, in the UK, the US, and other countries, national law prohibits the promotion of off-label uses of prescription drugs by pharmaceutical companies, which stand to benefit financially from off-label use through increased drugs sales. The primary rationale for banning off-label promotion is that it might encourage the widespread use of drugs in settings where they have not been rigorously tested, thereby exposing patients to uncertain benefits and possible adverse effects. In the US, the FDA regulates and prosecutes off-label promotion, but enforcement actions against companies can also be brought by federal and state prosecutors and private citizens. In the UK, the MHRA has delegated an important part of its responsibility for supervising off-label marketing to a self-regulatory body set up by the pharmaceutical industry—the PMCPA. Here, by reviewing off-label promotion rulings made by the PMCPA between 2003 and 2012, the researchers compare off-label promotion cases ruled on in the UK with whistleblower (company insider)-initiated cases from the US and shed light on the UK self-regulatory mechanism for detecting, deterring, and sanctioning off-label promotion.

**What Did the Researchers Do and Find?:**

The researchers identified 74 UK off-label promotion rulings over the ten-year study period involving 43 companies (including 19 that were ruled in breach more than once) and 65 drugs. They coded and analyzed each off-label promotion ruling using a typology (a classification according to general type) previously developed to analyze US whistleblower-initiated off-label promotion cases. Half of the rulings cited efforts to expand drug use to unapproved indications (for example, using a drug to treat all patients with MS rather than only patients with recent relapses); 39% and 38% of the rulings cited efforts to expand drug use beyond approved diseases and dosing strategies, respectively. The most commonly cited off-label promotional tactic was attempts to influence prescribers using advertisements and other print material; competing companies lodged 57% of complaints whereas prescribers (the prime target of off-label promotion) lodged only 22% of the complaints. Unlike US whistleblower complaints, which often alleged promotional tactics targeting consumers, payers and company staff, few UK rulings described practices targeting these classes of individuals. Finally, although several US whistleblower-initiated cases alleged multifaceted and covert marketing activities, the UK cases typically related to one or a few claims made in printed material.

**What Do These Findings Mean?:**

Because this study only describes PMCPA rulings and whistleblower-initiated US cases of off-label promotion, these findings provide an incomplete view of off-label marketing violations (some of which may not be deliberate) in the UK and US. The findings suggest that the UK self-regulatory approach, which relies mainly on complaints from company outsiders, is capable of detecting and dealing with instances of off-label promotion with high visibility (for example, advertisements). However, the UK self-regulatory approach may be less capable of uncovering complex marketing campaigns than the US government-led approach. The researchers suggest, therefore, that the UK authorities should consider the introduction of increased incentives and protections for whistleblowers and of US-style governmental investigation (the PMCPA rulings are based on complainant and company submissions alone) and that both the MHRA and PMCPA should strengthen their regulatory oversight of promotional material. Finally, prescribers—the main target of off-label promotion in the UK—should be encouraged to identify and report off-label promotion.

**Additional Information:**

This list of resources contains links that can be accessed when viewing the PDF on a device or via the online version of the article at http://dx.doi.org/10.1371/journal.pmed.1001945.

- The *PLOS Medicine* Research Article by Aaron Kesselheim and colleagues provides information about whistleblower-initiated cases on off-label promotion in the US
- Wikipedia provides information on prescription drugs, pharmaceutical marketing (mainly in the US), and off-label drug use (mainly in the US) (note that Wikipedia is a free online encyclopedia that anyone can edit; available in several languages)
- The US FDA Office of Prescription Drug Promotion aims to protect public health by assuring prescription drug information is truthful, balanced, and accurately communicated; the FDA’s Bad Ads Program aims to educate health care professionals about the role they can play in ensuring that drug advertising and promotion is truthful and not misleading
- Information on the UK regulatory framework is available from the MHRA; the MHRA website also includes information on advertising investigations by the MHRA.
- Information on the UK self-regulatory body, the PMCPA, is available; the PMCPA website includes information about the UK Code of Practice for medicines promotion
- Healthy Skepticism is an international non-profit membership association that aims to improve health by reducing harm from misleading health information

## Introduction

European Union (EU) law prohibits companies from promoting medicines off-label, that is, for a nonauthorized indication or in a nonauthorized form, strength, or dosage [1]. The primary rationale for the ban on off-label promotion is that promotion of pharmaceuticals whose effectiveness and safety has not been confirmed may entail serious risks to patients and unjustified cost to the health care system [2,3]. Moreover, off-label promotion may challenge the integrity of the medicines regulatory system by undermining the authority of regulators and discourage companies from conducting trials for new medicines or indications [4–6].

In the United Kingdom, as in other EU countries, provisions banning off-label promotion are incorporated in national law [7,8]. The Medicines and Healthcare products Regulatory Agency (MHRA) is responsible for enforcing marketing regulations, but the medicines regulator has delegated an important part of this responsibility to the UK industry trade groups [9,10]. To discourage illicit marketing, the industry trade groups have developed voluntary codes of practice administered by their own system of self-regulation [11]. Thus, in 1993 the Association of the British Pharmaceutical Industry (ABPI) established the Prescription Medicines Code of Practice Authority (PMCPA) as the quasiautonomous self-regulatory body responsible for administering the ABPI Code covering the marketing of prescription drugs. Whilst the MHRA considers a small number of allegations of illegal promotion each year, the majority of complaints are, therefore, administered by the PMCPA [10].

Whereas both the law and the ABPI Code prohibit off-label promotion, off-label prescribing is lawful (albeit controversial) and widespread—for example, in oncology [12–14], psychiatry [15,16], paediatrics [17–19], and palliative care [20,21]. Although some off-label prescribing is evidence based and/or unavoidable [22–24], studies also show that a significant amount of off-label use takes place without sufficient scientific evidence supporting this practice [2,25,26], and there is also evidence that off-label prescribing has caused serious harm to patients [27,28].

Despite acknowledged risks to patient safety, companies have a financial incentive to increase their market share by illicitly promoting off-label use of drugs [23,29,30], and research indicates that this promotional activity is effective in stimulating off-label prescribing [31,32]. To protect patients, it is therefore imperative that governments have robust enforcement systems in place to deter industry off-label promotion. However, scholarly analyses have highlighted the limitations of current legislative and regulatory frameworks for governing pharmaceutical promotion [5,10,33], and a recent, systematic analysis of 41 US whistleblower complaints found that some of the off-label marketing practices most commonly employed by companies may also be the most difficult for external regulatory bodies to detect and control [34]. Similarly, a US Government Accountability Office (GAO) investigation of the Food and Drug Administration’s (FDA’s) oversight of off-label promotion highlights the challenge of regulating off-label promotion that takes place in the context of Continuing Medical Education events or private meetings between physicians and company sales representatives—particularly when insufficient resources are devoted to monitoring and surveillance efforts [35].

Weaknesses in the FDA’s regulation and prosecution of off-label promotion in the US are to some extent offset by the existence of alternative mechanisms for prosecuting illegal industry marketing practices. Specifically, enforcement actions against companies can be brought by federal and state prosecutors and private citizens for violations of criminal and civil laws [5]. Prosecutions at the federal level are led by the Department of Justice (DOJ) and rest on extensive and complex investigations that typically span many years and involve examination of thousands of pages of company documents, detailed testimony, and reports [36,37]. In recent years, the majority of US federal prosecutions of pharmaceutical companies for off-label promotion have rested on whistleblower-initiated actions under the False Claims Act (FCA) [38]. Mello and colleagues argue that this litigation “has added a potent new dimension to the regulation of off-label promotion” [5]. In addition, extensive examination of company documents and witnesses in the course of DOJ and congressional investigations has provided an extraordinary insight into the complex range of marketing strategies and deceptive practices that companies engage in and the coordinated and planned nature of their campaigns [30,36–41].

In contrast to the growing awareness and increased understanding of the industry’s illicit marketing in the US, much less is known about off-label promotion in the UK (or the EU more broadly), despite the potential for significant harm to patients. Equally, there has been little consideration of the extent to which current institutional arrangements for regulating off-label promotion in the UK are effective in detecting, deterring, and sanctioning off-label promotion compared to the most commonly pursued enforcement strategies in the US. For example, previous research has shown that the self-regulatory system for detecting violations in the UK relies heavily on complaints from parties external to companies rather than internal whistleblowers [10]. Furthermore, the PMCPA is not an investigative body. Rather, PMCPA rulings are based on each party’s submissions, and the burden is on the complainant to show, on the balance of probabilities, that a breach of the ABPI Code has occurred [42]. Cases are determined relatively quickly, and the PMCPA will impose an administrative fine for breaches of the ABPI Code, not for violation of relevant UK or EU laws.

Given the diverse approaches taken in the UK and US in regulating pharmaceutical marketing—including a very significant difference in the level of resources invested in investigative and enforcement activities against illegal promotion—one might expect the kinds of cases brought under the respective systems to differ too. Yet, as indicated, there has been no systematic evaluation of the PMCPA’s regulation of off-label promotion. Nor has there been any analysis of the degree of overlap—if any—between PMCPA rulings and whistleblower-initiated cases prosecuted in the US under the FCA. Indeed, the ABPI has claimed that the US legal settlements are “simply not relevant to the UK market” [43].

This study begins to address these gaps by offering a structured review of all PMCPA rulings relating to off-label promotion over a 10-y period between 2003 and 2012. We compare the pattern of off-label marketing strategies and practices derived from UK cases (this study) with the pattern uncovered by the first structured review, undertaken by Kesselheim and colleagues, of off-label promotion lawsuits derived from whistleblower complaints in the US between 1996 and 2010 [34]. We also update their list of US whistleblower cases to include settled federal cases through June 2015 and investigate the degree of overlap between UK and US cases. Our objectives are to characterize the off-label promotion cases brought under the UK self-regulatory regime compared to the whistleblower-initiated federal cases and to shed light on the mechanisms for detecting, deterring, and sanctioning off-label promotion in the UK.

## Methods

### Cases and Violations

The PMCPA assembles individual complaints into cases that pertain to alleged breaches of the ABPI Code. Cases are subdivided into multiple matters for independent rulings, i.e., numerous matters may arise in a particular case, and each is considered independently [10]. The primary data for this study consist of publicly available PMCPA reports of breaches of §3.2 of the ABPI Code, which states that “the promotion of a medicine must be in accordance with the terms of its marketing authorisation and must not be inconsistent with the particulars listed in its summary of product characteristics” (SPC).

We identified cases involving one or more §3.2 breaches between 2003–2012 from quarterly PMCPA reports that include a summary of the outcomes for each case as well as individual case reports. The PMCPA reports cases in their final form, which means that all successfully appealed cases were excluded. We selected 2012 as the cutoff to ensure that we had included all cases for the last year, i.e., that no PMCPA cases were pending. Because §3.2 stipulates that promotion should be consistent with the SPC, and since the SPC includes additional information aside from the authorized uses of the drug, such as pharmacological properties, each case was reviewed independently by two authors (AV and SM) to ensure that it actually involved off-label promotion, defined as promotion for a nonauthorized disease, indication, or patient group, or in a nonauthorized form, strength, or dosage. The PMCPA highlights what it considers to be particularly serious violations by a simultaneous ruling of breach of §2 (promotion that “brings discredit to, and reduction of confidence in, the industry”), see [10]. We used §2 rulings as a signal of misconduct that might be especially grave.

### Complainants, Violating Companies, and Promoted Drugs

We collected information on complainants, violating companies, and promoted drugs from each case report [10]. Drugs were coded according to the consistent Anatomical Therapeutic Chemical (ATC) codes. Complainants were coded according to the following categories, which are used by the PMCPA in its yearly summaries: industry, health professional, MHRA, other organization, active monitoring by the PMCPA Director, and other (e.g., anonymous, member of public, industry employee/ex-employee). We also included the category “PMCPA Director: external complaint.” These are complaints nominally attributed to the PMCPA Director but not initiated following active monitoring. Instead, such complaints have usually emerged either in response to media criticism, from voluntary admissions by companies, or when the allegation is that a company has failed to comply with an undertaking that was given in relation to a previous ruling [10].

### Economic Sanctions

The PMCPA imposes administrative charges on violating companies. Until 2010, the charge per matter was £2,500 for ABPI member companies and £3,500 for nonmembers, which in 2011 was increased to £3,500 and £4,000, respectively. We used these figures to calculate the administrative charges for manufacturers associated with off-label promotion rulings. Companies can appeal the first ruling of the PMCPA, but there is an extra charge per unsuccessfully appealed matter. Since charges for processing appeals are unrelated to the violation, we did not include them in our cost calculation.

### Strategies and Practices of Off-label Promotion

After two authors (AV and SM) had independently verified the off-label promotion rulings, one of us (AV) conducted structured reviews of the off-label promotion cases and made a summary of each case and a preliminary manual (i.e., without qualitative data analysis software) coding of content [44] based on the typology developed by Kesselheim et al. [34] for their analysis of US whistleblower complaints. Using a standard coding methodology for abstracting information [45], the authors of that study identified two major descriptive domains pertaining to the alleged strategic goals of each off-label marketing scheme and the practices consistent with those alleged goals, respectively. The use of a previously established typology, rather than the creation of novel categories, enables comparison between rulings by the PMCPA and US whistleblower complaints. However, this also meant that not all categories abstracted from US whistleblower complaints were represented in the UK data. In addition to these previously established categories, we therefore extracted information from each case report on the communication channel(s) used by companies to promote off-label (e.g., journal ads) using basic content analysis [44]. Moreover, although we refer to companies’ strategies and tactics, it is important to note the possibility that some violations of the ABPI Code may have been unintentional.

The first descriptive domain described by Kesselheim et al. [34] consists of three nonmutually exclusive marketing strategies: (1) expansion to unapproved disease entities, i.e., disease entities distinct from those covered in the SPC; (2) expansion to unapproved variations of approved indications, i.e., different manifestations of the same disease or a different patient subgroup than for which a drug has marketing authorization; and (3) expansion to unapproved dosing regimens, i.e., use of doses or durations of treatment inconsistent with the SPC. To distinguish between expansion to unapproved disease entities and indications, respectively, we compared the promoted off-label use with the allowed on-label use as specified in PMCPA rulings and the SPC. For this, we used the International Statistical Classification of Disease and Related Health Problems (ICD) diagnosis codes [46]. The second major descriptive domain consist of four nonmutually exclusive practices that describe the targeted audience or the context of promotional activities: (1) prescriber-related practices, i.e., off-label promotion intended to influence prescribers; (2) consumer-related practices, i.e., off-label promotion to consumers; (3) internal practices, i.e., incentives and other practices directed at employees of the company, and (4) payer-related practices, i.e., practices aimed at encouraging insurers to pay for off-label prescription. Some categories also included one or more subcategories; for example, expansion to unapproved indications included the subcategory “different patient subgroups” [34]. The preliminary coding and summary of cases was confirmed by a second author (SM). In a few cases (*n* = 5; 7%), there was discrepancy between authors’ assessment. We solved discrepancies by comparing and discussing the results, after which we reached a consensus decision following a joint coding of the data.

### Overlap between PMCPA Rulings and Federal Whistleblower Cases in the US

A list of federal whistleblower cases until October 2010 was obtained from Kesselheim et al.’s analysis of whistleblower complaints [34]. We updated the list to include DOJ-settled whistleblower cases through June 2015 by conducting a search of DOJ press releases. We chose this cutoff rather than 2012, because FCA cases typically take many years to resolve, which means that cases settled after 2012 can relate to activities that took place in 2012 or prior. We crosschecked the final list with data compiled by Taxpayers Against Fraud, a nongovernmental organization that tracks federal fraud suits [47]. We considered US and UK cases to overlap when the allegations involved promotion of the same drug for the same off-label use. The degree of overlap was then determined from detailed readings of the DOJ press releases and linked documents and the PMCPA rulings.

## Results

### Off-label Promotion Rulings

**Fig 1:**
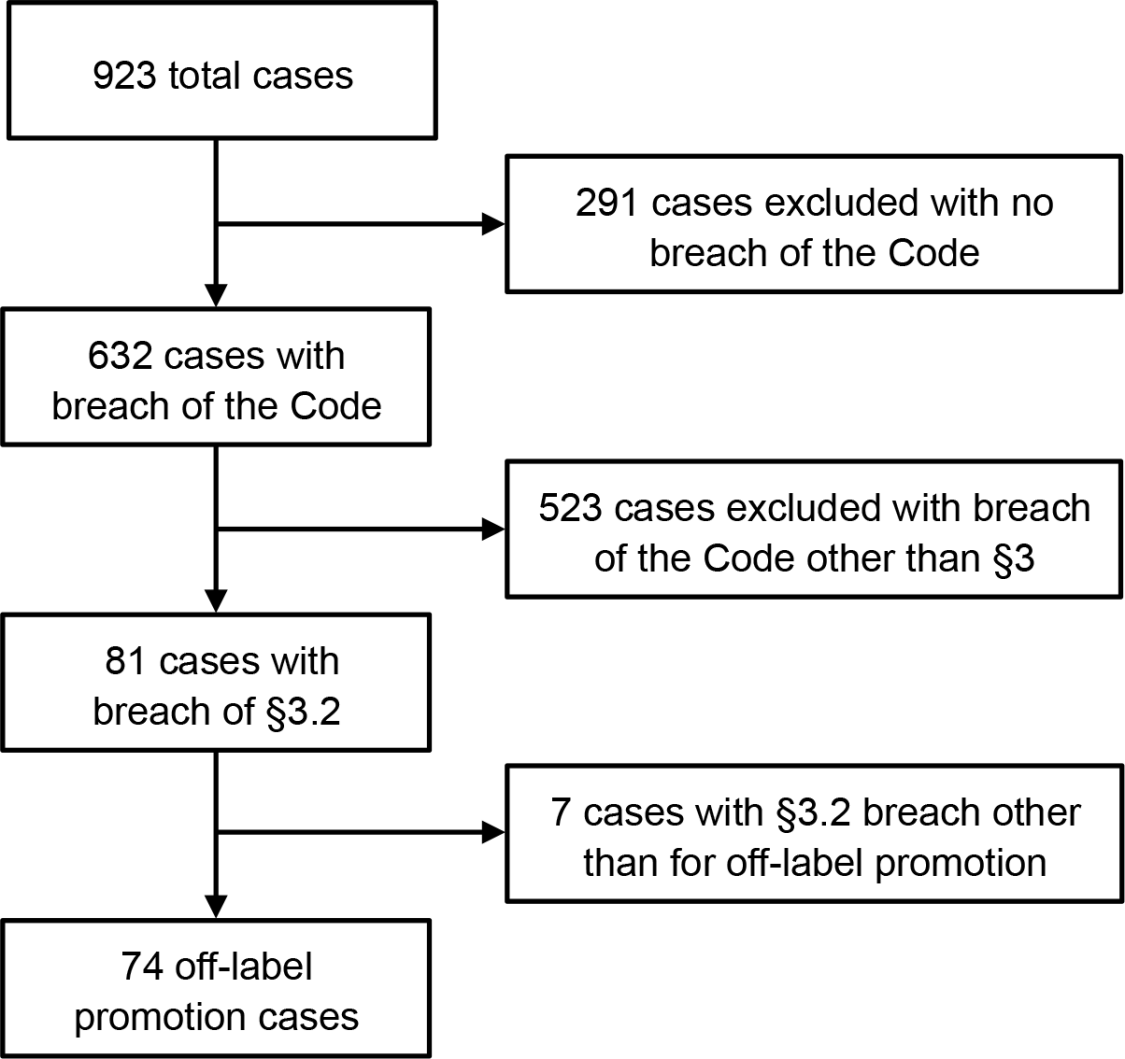
Flow diagram of selected cases.

**Fig 2:**
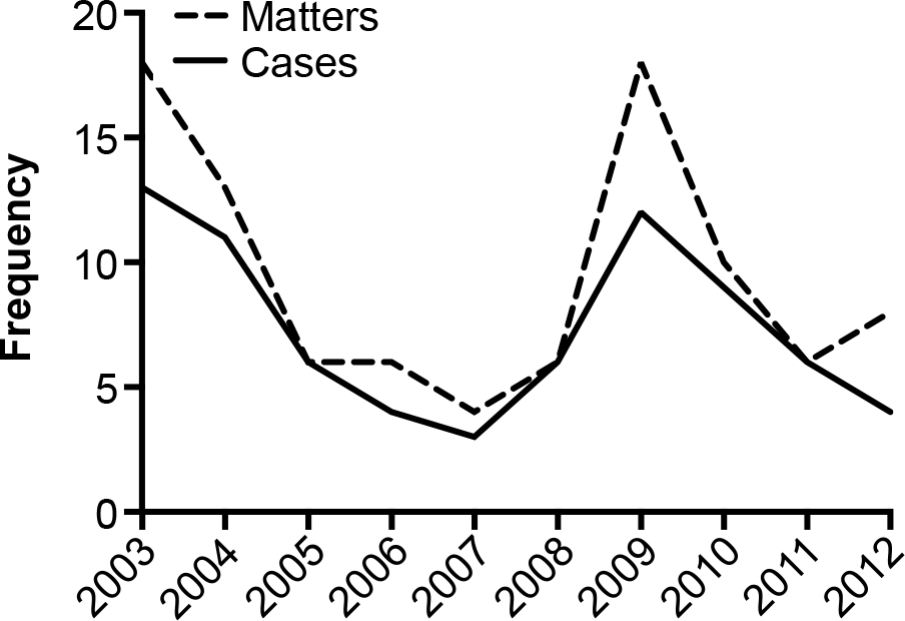
Off-label promotion rulings in the UK 2003–2012: Cases and matters in breach.

The study selection process is shown in Fig 1. From 2003–2012, the PMCPA ruled a total of 74 cases and 95 matters in breach for off-label promotion (S1 Table for list of cases). This amounts to 12% and 4% of total cases and matters ruled in breach, respectively. Off-label promotion rulings peaked in 2003 and 2009 (Fig 2). Ten rulings also included a breach of §2, i.e., were highlighted as particularly serious cases of misconduct by the PMCPA (Table 1). Off-label marketing violations regarded as particularly serious included failures to comply with an undertaking previously given, cases involving especially aggressive marketing, marketing posing a risk to patient safety, and marketing of a prescription-only drug to the public.

**Table 1:** Serious (§2) off-label promotion violation rulings, 2003–2012.

Case no.	Case summary	Drug class/ATC
1544/1/04	Napp marketed **OxyContin (prolonged release oxycodone)** in detail aid and leave piece^1^ with claims of “Straightforward dosing” and “5 mg starting dose in frail patients or those with renal or hepatic impairment.” However, the drug was contraindicated in patients with moderate to severe hepatic impairment or severe renal impairment. According to the ruling, claims gave the impression that patients with any degree of renal or hepatic impairment could have treatment initiated at 5 mg. This could lead to OxyContin use in patients for whom it was contraindicated and might therefore compromise patient safety.	Analgesics (N02)
1557/2/04	At a scientific meeting, Aventis promoted an off-label use of **Taxotere (docetaxel)** by soliciting requests from health professionals for a publication that discussed unlicensed use of Taxotere in combination with carboplatin. Dissemination of the publication had previously been found to breach the Code. Aventis was therefore in breach of an undertaking.	Antineoplastic agents (L01)
1708/5/05	After reports of adverse effects in ongoing clinical trials for a competitor’s MS (multiple sclerosis) drug, Serono sent company-produced clinical guidelines with a cover letter signed by Serono’s medical director for northern Europe to trial investigators encouraging them to switch patients over to Serono’s drug **Rebif (interferon beta-1a)**. Provided information implied that Rebif could be used to treat all MS patients, but Rebif was indicated for the treatment of MS with two or more relapses within the last 2 y. This was considered a particularly serious matter “where it appeared aggressive marketing had been undertaken with disregard of the Code.”	Immuno-stimulants (L03)
2008/6/07	ProStrakan reused an advertisement with a claim that **Rectogesic (glyceryl trinitrate)** could heal chronic anal fissures when it was only licensed for relief of pain associated with chronic anal fissures. The claim had previously been ruled in breach and ProStrakan was thus in breach of an undertaking.	Vasoprotectives (C05)
2125/5/08	Advertisement by Takeda in Diabetologia claimed that “there are no long-term cardiovascular concerns regarding the use of **Actos (pioglitazone)**.” However, there was no mention that Actos was contraindicated in patients with cardiac failure or a history of cardiac failure or might cause fluid retention, which might exacerbate or precipitate heart failure. The MHRA had found Takeda to have breached advertising regulations for a similar advertisement before, and had also asked Takeda to issue a corrective statement. The PMCPA expressed its concern for patient safety.	Diabetes drugs (A10)
2330/7/10	In a poster session at the British Pain Society meeting, Grünenthal promoted **Versatis (lidocaine)** with the following claim: “Conclusion, Versatis has an ‘off-label’ use for the symptomatic relief of localised neuropathic pain, and could provide a substantial saving to the local health-economy.”	Anaesthetics (N01)
2383/2/11	Bayer promoted **Yasmin (ethinylestradiol and drospirenone)** with the following claim: “Yasmin. It’s for more women than you might imagine.” The product logo to the right of the claim at issue included the strapline “Contraception and more.” A bullet point stated that Yasmin had been shown to have a beneficial effect versus baseline on acne, fluid retention, hirsutism, and premenstrual symptoms. These were not licensed indications but instead included possible adverse reactions listed in the SPC.	Sex hormones and modulators of the genital system (G03)
2404/5/11	Boehringer Ingelheim issued a press release on **Pradaxa (dabigatran)** that formed the basis for articles in the consumer press referring to Pradaxa use for stroke prevention, an unlicensed indication. Boehringer Ingelheim had promoted a prescription-only medicine to the public in an indication that was not yet licensed, the PMCPA concluded.	Antithrombotic agents (B01)
2435/8/11	Chiesi invited UK health professionals to a symposium at which **Fostair (beclometasone and formoterol)** was promoted for chronic obstructive pulmonary disease (COPD) when only licensed in the UK for the regular treatment of asthma. Chiesi had previously been ruled in breach for Fostair promotion off-label at a meeting of the British Thoracic Society. Chiesi had thus failed to comply with a previous undertaking.	Drugs for obstructive airway diseases (R03)
2506/5/12 and 2507/5/12	Lilly and Daiichi-Sankyo marketed **Efient (prasugrel)** with a leave piece^1^ that promoted use beyond the maximum licensed duration of treatment. The SPC stated, “Treatment of up to 12 mo is recommended, unless the discontinuation of Efient is clinically indicated …”, but the leave piece promoted off-label use for 15 mo. The leave piece also included various misleading statements and misrepresentation of safety data. A breach of §2 was ruled in relation to the leave piece as a whole.	Antithrombotic agents (B01)

^1^ Leave piece refers to print marketing material that is left behind during a sales visit.

Companies collectively paid around £260,000 (€360,000) in administrative charges for off-label promotion over the study period. In total, 43 companies were ruled in breach at least once. Almost half of these companies (*n* = 19) were ruled in breach more than once over the 10-y period, and ten companies were ruled in breach three or more times (S2 Table). Sixty-five drugs that fell into 35 ATC classes were represented in rulings, with diabetes drugs being most prevalent, followed by drugs for obstructive airway diseases (S3 Table).

More than half (*n* = 42; 57%) of cases were initiated after complaints from rival companies. Health professionals initiated 16 cases (22%). The PMCPA Director initiated one case following active monitoring (1.4%). In addition, eight cases (11%) were nominally attributed to the PMCPA Director but emerged after voluntary admissions by companies (*n* = 4) or media reports (*n* = 1) or involved breaches of undertaking (*n* = 3). Six (8%) complaints came from other individuals—two anonymous company representatives, one company employee, and three anonymous complainants—and one (1.4%) came from an organization. No case was initiated following complaint from the MHRA.

### Off-Label Marketing Strategies

Our analysis considered three nonmutually exclusive strategic goals underlying companies’ off-label marketing activities. Table 2 reports on the number of cases that fell into each category. To allow for comparison to the previous analysis of US whistleblower complaints, we have also included data reported in that study [34].

**Table 2:** Frequency of off-label marketing strategies and practices reported in PMCPA rulings (2003–2012) and US whistleblower complaints (1996–2010).

Descriptor	UK Cases, n/N (%)	US Whistleblower Complaints, n/N (%)^1^
**Off-label marketing strategies**		
Expansion to variation of approved indication	37/74 (50%)	22/41 (54%)
*Different patient subgroup*	*20/37 (54%)*	*10/22 (45%)*
Expansion to different disease entity	29/74 (39%)	35/41 (85%)
*Similar symptoms*, *different disease*	*15/29 (52%)*	*17/35 (49%)*
Expansion to variation of approved dosing schedule	28/74 (38%)	14/41 (34%)
**Off-label marketing practices** ^**2**^		
Prescriber-related	72/74 (97%)	41/41 (100%)
Internal practices	2/74 (3%)	37/41 (90%)
Payer-related	2/74 (3%)	23/41 (56%)
Consumer-related	3/74 (4%)	18/41 (44%)

^1^ Data adapted from Kesselheim et al. [34].

^2^ US whistleblower complaints report specific practices not present in UK rulings, e.g., ghost-writing, giving free samples to prescribers, and discussion with prescribers about how to ensure reimbursement (not shown). UK rulings mostly report dissemination of print material: see Table 3.

#### Expansion to unapproved indications

The most commonly described strategy involved attempts to expand medicines use to unapproved indications, i.e., variations of the approved condition (37/74, 50% of cases). One example concerned Rebif (interferon beta-1a), licensed for MS patients with two or more relapses within the last 2 y but marketed as a treatment for all MS patients (case no. 1708/5/05; see Table 1). In many instances, the drug was promoted for unauthorized patient subgroups (20/37, 54%). For example, the diabetes drug Actos (pioglitazone) was marketed with a claim that it could be used without long-term cardiovascular concerns; however, this was inconsistent with the contraindication in patients with, or with a history of, heart failure (no. 2125/5/08; see Table 1).

#### Expansion to unapproved disease entities

The second most prevalent described strategy was to promote drugs for unauthorized diseases (29/74, 39%). For example, Bondronat (ibandronate), a drug indicated for the treatment of tumour-induced hypercalcaemia, was promoted at a regional breast cancer meeting with an article reprint referring to uses in normocalcaemic women (no. 1526/10/03). In many examples of this kind of expansion strategy, the drug was promoted for treatment of similar symptoms across different disease conditions (15/29, 52%). For example, the obstructive airway disease drug Fostair (beclometasone and formoterol) was promoted for use in COPD, whereas the medicine was approved only for the regular treatment of asthma (no. 2435/6/12; see Table 1).

#### Expansion to unapproved dosing strategies

Almost as frequent as described attempts to expand beyond approved disease entities was the strategy of expansion to unapproved dosing schedules (28/74, 38%). For example, prescribing information provided for Cialis (tadalafil) to treat erectile dysfunction stated “Maximum dosing frequency, once per day,” but failed to refer to the fact that daily dosing was strongly discouraged in the SPC (no. 1506/8/03). It could also mean that a drug was promoted beyond the maximum licensed duration. For example, the antithrombotic agent Innohep (tinzaparin) was promoted for long-term use in cancer patients even though the data relating to side effects and safety in the SPC was limited to short-term use (no. 2209/2/09).

### Off-label Marketing Practices

The most frequently described promotional practice involved attempts to influence prescribers (*n* = 72, 97%) (Table 3). However, many of the prescriber-related practices reported by US whistleblowers, e.g., ghost-writing, provision of free samples, and discussion with prescribers on how to ensure reimbursement [34], were not described in PMCPA rulings. Moreover, few PMCPA rulings related to practices targeting consumers, payers, or company staff.

**Table 3:** Off-label communication channels reported in PMCPA rulings 2003–2012.

Off-label Communication Channel	Cases n/N (%)
**Prescriber-Related**	***n* = 72**
Print material^**1**^	70/72 (97%)
*Journal advertisement*	*21/70 (30%)*
*Leave piece* ^**2**^	*14/70 (20%)*
*Letter*	*13/70 (19%)*
*Detail aid*	*8/70 (11%)*
Journal publication	7/72 (10%)
Face-to-face	11/72 (15%)
*Promotional stand at medical gathering*	*7/11 (64%)*
*Company presentation*	*4/11 (36%)*
Internet	5/72 (7%)
*E-mail*	*4/5 (80%)*
*Website*	*1/5 (20%)*
**Internal Practices**	***n* = 2**
E-mail	1/2 (50%)
Telephone conference	1/2 (50%)
**Payer-Related**	***n* = 2**
Advertisement	1/2 (50%)
Budget impact model	1/2 (50%)
Poster	1/2 (50%)
**Consumer-Related**	***n* = 3**
Website	1/3 (33%)
Press release	2/3 (67%)

^1^ For print material to prescribers, communication channels cited in *n* > 5 cases are included.

^2^ Leave piece refers to print marketing material that is left behind during a sales visit.

#### Prescriber-related practices

Instead, most UK rulings described companies’ use of print material (70/72, 97%), for example, advertisements (21/70, 30%), to promote off-label to prescribers (Table 3). For example, the anaesthetic Versatis (lidocaine medicated plaster) was promoted in an ad in the British Medical Journal with the claim “New for burning, shooting, stabbing pains” implying that Versatis was licensed to treat any such pain irrespective of its origin when in fact the drug was only licensed for post herpetic neuralgia (no. 1960/2/07; Table 1). Off-label use was also sometimes encouraged by disseminating journal publications covering the unapproved use (7/72, 10%). For example, marketers disseminated a review article from a stand at a conference that discussed ongoing or planned clinical studies for their company’s immunosuppressant drug Myfortic (mycophenolate sodium) in different patient populations and treatment regimens including withdrawal or avoidance of steroids for which the drug had no license (no. 1851/6/06). Communication “face-to-face” was also described (11/72, 15%) as, for instance, in the Fostair ruling described in Table 1 (no. 2435/6/12).

#### Internal, payer-related, or consumer-related practices

There were two descriptions of internal practices (2/74, 3%). In one case, an anonymous company representative had been encouraged during an internal telephone conference to promote the dermatological antibiotic Aldara (iniquimod cream) off-label to plastic surgeons to shrink lesions prior to surgery when the drug was only indicated for the topical treatment of small superficial basal cell carcinomas (no. 2102/3/08). Payer-related practices were reported in two cases (2/74, 3%). An example detailed in Table 1 pertained to promotion of the anaesthetic Versatis (lidocaine medicated plaster) in a conference poster (no. 2330/7/10). Three rulings described off-label marketing to consumers (3/74, 4%). In one case, the company had used a medical journal ad to refer health professionals, and encouraged health professionals to refer patients, to a patient group website that included a newsletter giving information about the use of the anti-Parkinson drug Requip (ropinirole) for restless leg syndrome, an unlicensed indication (no. 1801/2/06). In another case, the antithrombotic agent Pradaxa (dabigatran) was promoted in a press release that formed the basis for articles in the consumer press referring to its use for stroke prevention, an unlicensed indication (no. 2404/5/11; see Table 1).

### Overlap with US Whistleblower Cases

Eight rulings (11%) pertaining to six drugs described promotion of the same drug for the same off-label use as was alleged by whistleblowers in DOJ-settled cases (Table 4). In one case, (Versatis/Lidoderm) the company marketing the drug differed in the two countries because of licensing arrangements. However, while the UK cases typically related to only one or a few claims made in printed promotional material, several complaints in the US alleged multifaceted and covert marketing activities. An example is the promotion of antipsychotics for use in nonschizophrenic psychotic disorders where US lawsuits revealed highly orchestrated sales operations, such as paying speaker fees to doctors to influence their prescribing or having a sales force tasked with promoting off-label uses. In contrast, the UK rulings—involving the same companies marketing the same drugs—referred only to claims and images in single adverts. Another alleged example of multifaceted and covert marketing in the US involved promotion of Botox where, among other things, the company allegedly had directed physician workshops and dinners focused on off-label uses, paid doctors to attend “advisory boards” promoting off-label uses, and created a purportedly independent online education organization to stimulate off-label use.

**Table 4:** UK and US off-label promotion cases involving the same drug for the same off-label use.

Drug	Company	Off-label Use	UK Case Summary	Year	Charge	US Case Summary^1^	Year	Settlement^2^
Aranesp (darbepoetin alfa)	Amgen	Unauthorized dosing regiment	Aranesp was approved for use once a week. However, advertisement quoted a paper entitled “Darbepoetin Alfa Administered Every 2 Weeks Alleviates Anemia in Cancer Patients Receiving Chemotherapy.” The implication of using Aranesp every 2 wk was that the initial dose would be higher. (no. 1554/2/04)	2004	£2,500	From 2002 until 2007, Amgen allegedly promoted Aranesp for (1) unapproved dosing regiments and (2) unapproved indications. Regarding the former, the complaint accused Amgen of building the Aranesp commercial strategy around a less frequent dosing schedule but with doses that were two to four times larger than approved.	2012	US$762 million^3^
Seretide (UK), Advair (US) (fluticasone/salmeterol)	GlaxoSmithKline	Mild COPD	Seretide was licensed in a restricted group of COPD patients, i.e., those with severe disease and a history of repeated exacerbations, who had significant symptoms despite regular bronchodilation. Furthermore, only one of the six Seretide formulations was so licensed. Claim in detail aid and journal ad, “Seretide in COPD,” without qualification implied that all formulations of Seretide could be used in any patient with COPD and this was not so. (no. 1551/2/04)	2004	£2,500	GSK allegedly promoted Advair off-label (1) for mild COPD and (2) as first-line therapy in mild asthma. Regarding the former, the complaint accused GSK of building the Advair commercial strategy around its “Only 1” campaign (July 2008–June 2010), which encouraged prescribing to patients who had had only one exacerbation. GSK allegedly made false and misleading statements about relevant treatment guidelines, trained representatives to lie about the guidelines, and gave representatives buttons to wear with the phrase “only one.”	2012	US$25 million^4^
Abilify (aripiprazol)	Otsuka (UK, US) Bristol-Myers Squibb (UK)	Nonschizophrenic psychotic disorders	Abilify was indicated for schizophrenia. Leave piece announcing launch stated “Highly effective symptom control in acute psychosis” and “Abilify helped to control the symptoms of acute psychosis as early as week 1.” However, schizophrenia is only one of the very many causes of acute psychosis, e.g., mania, depression, and drug abuse. (no. 1623/8/04 and 1624/8/04)	2004	£2,500	Allegations that, from 2002 through the end of 2005, Otsuka promoted Abilify off-label for (1) paediatric use and (2) to treat dementia-related psychosis. Regarding the latter, the complaint accused Otsuka representatives of participating in a specialized long-term care sales force that called almost exclusively on nursing homes, where dementia-related psychosis is far more prevalent than schizophrenia or bipolar disorder for which the drug was licensed.	2008	US$4 million
Risperdal (risperidone)	Johnson & Johnson (Janssen)	Children	J&J subsidiary Jansen issued advertisement featuring a lone female figure in a playground walking away from a trail of articles that included a doll, photograph album, wedding veil, handbag, and toothbrush. The impression that the figure had possibly once owned the articles on the ground was compounded by the adjacent text “Prescribe early, because what she loses, she could lose forever.” The statement “Prescribe early” implied that the figure in the photograph was a young person. The Risperdal SPC stated that the product had not been studied in children or adolescents younger than 18 y. (no. 2059/10/07)	2007	£2,500	From 1999 through 2005, Janssen allegedly promoted Risperdal for use in children, individuals with mental disabilities, and elderly dementia patients. J&J and Janssen allegedly knew that Risperdal posed certain risks to children, including risk of hormonal disturbances. Nonetheless, one of Janssen’s Key Base Business Goals was to grow and protect the drug’s market share with child/adolescent patients. The FDA repeatedly warned the company against promoting it for use in children. Janssen allegedly paid speaker fees to doctors to influence them to write prescriptions and told these doctors that if they wanted to receive payments for speaking, they needed to increase their Risperdal prescriptions.	2013	US$2.2 billion
Versatis/Lidoderm (lidocaine)	Grünenthal (UK), Endo (US)	Non-postherpetic neuralgia (PHN)	(1) Ad in the BMJ with claim “New for burning, shooting, stabbing pains” implied that Versatis was licensed to treat any such pain irrespective of its origin, when in fact the drug was only licensed for PHN. (no. 1960/2/07). (2) See Table 1 case no. 2330/7/10.	2007, 2010	£2,500, £2,500	From March 1999 through December 2007, Endo promoted Lidoderm for use in the treatment of non PHN-related pain. Allegations that sales managers provided instruction to sales representatives concerning how to expand sales conversations with doctors to off-label uses. Endo admitted that it intentionally promoted Lidoderm to health care providers for unapproved indications.	2014	US$193 million
Botox (botulinum neurotoxin)	Allergan	Headache (HA), etc.	(1) Product monograph and objection handler contained claim that Botox was “… approved in over 70 countries, with 20 licensed indications.” The PMCPA considered that although both documents listed the six indications approved in the UK, reference to the 20 licensed indications worldwide might solicit questions about indications not licensed in the UK. (no. 2215/3/09). (2) A survey headed “Neurology Pharmaceutical Survey” together with a check for £35 was sent by a market research agency. Six of the 22 questions referred to the use of botulinum injections for the treatment of primary HA or migraine, which were off-label uses. The PMCPA considered that the survey would stimulate interest in the off-label use of Botox. Upon reading the report, the MHRA concluded that the survey and associated payment breached advertising regulations by promoting Botox off-label and offering doctors a prohibited benefit. Allergan agreed to issue corrective statement to recipients of survey and check. The MHRA deemed it in public interest not to pursue doctors who had accepted the payment in good faith but asked them to donate the money to charity, (no. 2274/10/09).	2009, 2009	£5,000, £2,500	Allergan plead guilty to allegations of off-label promotion for HA, pain, spasticity, and juvenile cerebral palsy. The allegation was that Allergan exploited its on-label cervical dystonia (CD) indication to grow off-label pain and HA sales. In 2003, Allergan developed the “CD/HA Initiative” as a “rescue strategy” in the event of negative results from its clinical trials to ensure continued expansion into the pain and HA markets. As part of this initiative, Allergan claimed that CD was “underdiagnosed” and possible to diagnose based on HA and pain symptoms, even when the doctor “doesn’t see any.” Allergan’s marketing tactics also included calling on doctors who typically treat patients with off-label conditions and assisting doctors in obtaining reimbursement for off-label uses. Allergan held workshops to teach doctors and their office staffs how to bill for off-label uses, conducted detailed audits of doctors’ billing records to demonstrate how they could make money by injecting Botox, and operated the Botox Reimbursement Hotline, which provided free on-demand services to doctors for off-label uses. Allergan also lobbied government health care programs to expand coverage for off-label uses, directed physician workshops and dinners focused on off-label uses, paid doctors to attend “advisory boards” promoting off-label uses, and created a purportedly independent online education organization to stimulate off-label use.	2010	US$600 million

^1^ Summaries based on DOJ press releases: http://www.justice.gov/.

^2^ May include both civil settlements under the FCA and criminal settlements. May also include settlements for illicit activities other than off-label promotion.

^3^ The settlement also included illicit promotion of other drugs.

^4^ Settlement for COPD part. Settlement for Asthma part was US$686 million.

## Discussion

To our knowledge, this is the first study that systematically investigates evidence of off-label promotion in a European country. Our analysis considered 74 cases of off-label promotion involving 43 companies and 65 drugs and revealed a high occurrence of repeat violations over the 10-y period. Almost half of the companies in the sample were found to have promoted products off-label more than once, and about one-fourth were ruled in breach three or more times.

The analysis pointed to various nonmutually exclusive off-label marketing strategies and practices. With respect to strategies, 50% of rulings cited efforts to expand drug use to unapproved indications, and roughly 40% cited efforts to expand beyond approved disease entities and dosing strategies, respectively. With respect to practices, the centerpiece of off-label promotional tactics reported in PMCPA rulings involved attempts to influence prescribers, most typically via print material such as journal ads. Despite the fact that rulings described prescribers as the prime targets of off-label promotion, it was competing companies that lodged the majority of complaints. This finding points to the need for UK physicians to be attentive to, and increasingly report, off-label promotion.

We also found that the pattern of off-label marketing practices reported in PMCPA rulings differed from the pattern abstracted from US whistleblower complaints [34]. One set of differences relates to the paucity of practices targeting consumers, payers, and company staff in the UK sample. Such differences could reflect actual divergences in industry marketing between the countries, which in turn might be related to national differences in the legal, regulatory, and health care organizational contexts in which drug companies operate. For example, direct-to-consumer advertising of prescription medicines is illegal in the EU [48], which means that fewer opportunities probably exist for off-label promotion directly to UK consumers. Similarly, the many payer-related practices in the US sample might relate to the fact that in the US, health care providers certify to third-party insurers that a particular prescription was needed to get the health care claim paid [34].

Another set of differences was highlighted by our qualitative review of PMCPA rulings and DOJ press releases and relates to the scope and complexity of the corporate activity uncovered by US enforcement actions compared with the kinds of activity reported to the PMCPA. Activity uncovered by US DOJ investigations typically involved a range of diverse, yet carefully coordinated, corporate practices aimed at expanding sales through off-label use of drugs, including attempts to promote more than one off-label use. UK allegations, in contrast, described a much more restricted range of promotional activities and typically referred to a single advertisement, or other printed material, focused on a single off-label use. This finding held true even in cases in which the same transnational companies were discovered promoting the same drug product for the same off-label use in the UK and the US.

This raises the possibility that another explanation for the divergent UK–US patterns of detected violations lies in differences in the types of complainant and in the nature and extent of investigations pursued. Crucially, US complainants are whistleblowers that offer first-hand testimonies and documentation of company practices [5,34,39,40]. Complainants’ allegations are in turn subject to wide-ranging and prolonged investigations by the DOJ that will involve, for example, in-depth interrogation of complainants, subpoenas for documents or electronic records, witness interviews, consultations with experts, and sometimes search warrants to obtain further evidence. In the UK, the majority of complaints are lodged by individuals or organizations from outside the violating company, which means that evidence of internal practices and pressures on company staff will be rare. Indeed, the majority of allegations considered by the PMCPA involve promotional material with high-to-medium visibility and the greatest risk of detection [5,35]. Instructively, the two UK rulings pertaining to internal company practices emerged from complaints by anonymous employee whistleblowers. In addition, and compared to the extensive investigations undertaken by the US DOJ, regulatory scrutiny by the PMCPA lacks breadth and depth. The Panel does not regularly consider evidence beyond that submitted by the parties to the case [42], let alone undertake the type of investigation that would allow it to uncover sustained and complex off-label promotional campaigns of the kind described in the US cases [49].

Where other factors (such as diverse health care organizational contexts) are unlikely to account for differences in the pattern, range, and seriousness of the promotional activities discovered, a plausible alternative explanation is that the US legal and regulatory environment is more effective at detecting and interrogating off-label promotion than the UK’s self-regulatory system and provides far greater insight into the nature and extent of illegal corporate activity. Such insights are crucial for informing the imposition of appropriate sanctions and for the development of more effective regulatory responses to protect public health [34,36].

### Implications for Policy and Debates

As with previous studies of pharmaceutical promotion, this study raises critical questions regarding the impact of different regulatory arrangements in deterring illicit marketing [10,50–52]. In view of the fact that a significant portion of companies were found to have repeatedly breached the ABPI Code related to off-label promotion, it is reasonable to consider whether the UK self-regulatory system could be strengthened to better protect public health. Successful deterrence is based in the first instance on a credible threat of detection, which in turn depends on effective regulatory monitoring and surveillance and sufficient investigative capacity. A weakness of the UK regulatory apparatus, therefore, is that neither the PMCPA nor the MHRA—unlike the FDA [35] and the Swedish self-regulatory authority [10]—require companies to routinely submit post publication promotional material for review, even though this is clearly an important mechanism for detecting violations. For example, between 2003–2008, the FDA identified 31 cases of off-label promotion, corresponding to 74% of off-label promotion cases administrated by the FDA, through its screening of submitted promotional material [35]. Similarly, of all marketing violations reported by the Swedish self-regulatory authority, about 50% are detected through this mechanism [10]. We would recommend that both the MHRA and the PMCPA strengthen their regulatory oversight of published promotional material by requiring companies to submit all post publication promotional material for review. To increase the transparency and accountability of regulatory oversight, we also suggest making the submitted promotional material publicly available upon request.

Insights into the nature and scope of off-label promotion gleaned from US whistleblower complaints confirm that even with increased regulatory capacity, much illegal marketing will be difficult to detect and that discovery of the least visible activity will depend on reports from those with direct knowledge of it [34]. As noted above, a key tool in the US arsenal is the whistleblower provisions under state and federal FCAs [5,30,34,36,38]. Whilst some protection is afforded whistleblowers in the UK under the Public Interest Disclosure Act 1998, this legislation fails to provide the financial incentives (or financial protections) offered under the US FCA, which may explain the relatively low number of employee complaints in the UK sample. Greater incentives and protections for whistleblowers could result in increased exposure of less visible company practices to promote off-label use of drugs, where these exist.

Without improved investigative capacity, however, greater reliance on whistleblowers many not be enough to enhance the ability of regulatory bodies to uncover the full range of companies’ illegal marketing activities. Whilst the speed with which the PMCPA is able to administer cases and reach decisions is a positive feature of the self-regulatory system, the PMCPA lacks the investigative powers to discover the kind of carefully orchestrated company campaigns that DOJ investigations have revealed. Moreover, it is extremely rare for the MHRA to undertake investigations, or even further action, following PMCPA findings. We are aware of only two cases in our sample in which PMCPA off-label promotion rulings and related publicity prompted further action by the MHRA [53,54]. One case concerned Allergan offering doctors a prohibited benefit in the context of Botox off-label promotion and is detailed in Table 4. The other case involved Boehringer Ingelheim disregarding MHRA advice to remove information about Pradaxa (dabigatran) trials outside the authorized indications in promotional material. To strengthen the capacity of regulatory bodies to uncover relatively complex and hidden off-label promotional practices, the UK Government should increase incentives and protections for whistleblowers and encourage US-style investigation of allegations.

The deterrent capability of regulatory systems also depends on the existence of effective sanctions. The PMCPA collects administrative charges from violating companies, which it uses to finance self-regulation [10]. We estimated that companies over the study period jointly paid around £260,000 (€360,000) to the PMCPA for off-label promotion. Administrative charges in the UK do not reflect the seriousness of company breaches, nor are they designed to harm corporations financially. In fact, charges for violations are typically less than a company would pay for a single print advertisement [55]. It is highly unlikely, then, that PMCPA charges serve any kind of deterrent function. According to the PMCPA, the most important sanction available to the UK self-regulatory body to discourage violations is, rather, adverse publicity [56]. In serious cases, the PMCPA may issue a public reprimand or order companies to issue a corrective statement, and breaches of §2 (i.e., promotion that “brings discredit to, and reduction of confidence in, the industry”) are always publicized through advertisements placed in the professional press [10]. In very severe cases, the PMCPA may report the company to the ABPI, which may then temporarily suspend the offending company from the trade group. However, it is unclear whether this publicity poses a significant reputational risk to companies. Advertisements provide scant detail relating to companies’ activities and refer to breaches of the ABPI Code rather than criminal offences, and it is instructive that advertised cases have failed to generate anywhere near the level of adverse publicity that has been generated by legal actions in the US. There is a need for the UK government and regulatory bodies to develop a range of sanctions that can more effectively deter and control industry off-label promotion [10].

Finally, the current coregulatory arrangement in the UK between the PMCPA and MHRA appears to have resulted in a situation where companies that may have committed criminal offences under UK and EU laws by promoting their drugs off-label are almost never subject to statutory controls or enforcement action. Despite evidence of repeated violation, including cases that the PMCPA judged particularly serious, we found no evidence that any off-label promotion cases had been referred to the MHRA for consideration for prosecution. Companies found to have committed serious violations should also be prosecuted under the relevant legislation and, if convicted, subject to meaningful and proportionate sanctions [57–59]. Although intensive investigations and legal proceedings are expensive, US states and the federal government have more than recouped the costs of such actions through the fines levied on offending companies [38].

Since most off-label marketing in the UK and US appears directed at prescribers, they are both an important potential source of information and a potential safeguard against illegal promotion [34]. Currently, however, physicians rarely report instances of off-label marketing to the PMCPA, and one reason for this may be lack of awareness of licensed indications or of the existing evidence base for prescription medicines [60,61]. While our discussion has mainly focused on “downstream” interventions to control industry off-label promotion, preventive interventions targeted at raising awareness amongst physicians, such as government-sponsored “academic detailing” or educational workshops for medical students, have been shown to improve the quality of prescribing and might also increase physician awareness of, and intolerance towards, illegal off-label marketing [62,63].

### Strengths and Limitations

A strength of this study is that it was based on final PMCPA rulings rather than on allegations. The evidence for off-label promotion is therefore strong, although it cannot be assumed that every violation of the ABPI Code was deliberate. On the other hand, the use of a coding scheme developed for US whistleblower complaints may have led us to overstate similarities between countries, although this was counterbalanced by our review of PMCPA rulings and DOJ press releases, which highlights important qualitative differences. In addition, our analysis offers an incomplete view of the off-label marketing violations uncovered in both countries. First, some allegations in the UK are considered by the MHRA rather than by the PMCPA [10]. However, the total number of cases is relatively small. By scrutinizing publicly available reports of advertising investigations on the MHRA webpage for the period 2003–2012, we identified nine rulings pertaining to off-label promotion, of which five involved ABPI members and three overlapped with PMCPA cases. This compares to the 74 rulings considered by the PMCPA over the same period. Whilst it would, in theory, have been possible to include these nine MHRA rulings in the study, published MHRA reports (unlike PMCPA reports) do not provide sufficient detail to undertake the kind of structured review or qualitative analysis undertaken here. Second, we did not include off-label promotion cases handled directly by the FDA [35]. Although a comparison of PMCPA and FDA regulation of off-label marketing is an important area for future research, our objective in this study was to compare off-label promotion cases brought under the UK self-regulatory regime with whistleblower-initiated federal cases in the US to explore the outcomes of two very different regulatory approaches. Third, because we considered US cases prosecuted at the federal level, we may have overlooked some off-label promotion cases prosecuted at the state level [38]. Fourth, our list of federal whistleblower cases may be incomplete; indeed, to our knowledge, there is no comprehensive list of federal enforcement actions against pharmaceutical companies. Fifth, we based in part our cross-country comparison on information in DOJ press releases that, although detailed, may not provide a comprehensive list of alleged practices. Finally, because off-label promotion lawsuits typically take years to resolve, there could be additional cases pending in the US. Future research should address these limitations to offer a more complete view of the strengths and weaknesses of various approaches to the regulation of off-label promotion.

### Conclusion

The UK self-regulatory authority is capable of dealing relatively quickly with instances of off-label promotion with high visibility such as advertising. However, relative to the US government-led approach, our study provides evidence of the limited capacity of the UK’s self-regulatory arrangements to uncover complex marketing campaigns that remain concealed to company outsiders. To detect and take appropriate enforcement action against such campaigns in the UK, should they exist, authorities would need to introduce increased incentives and protections for whistleblowers combined with US-style governmental investigations and meaningful sanctions. Since much off-label promotion in the UK appears directed at prescribers, it is important that health professionals are attentive to and increasingly report instances of off-label promotion. Furthermore, cross national comparative research is needed to shed light on different national systems for regulating off-label promotion, including research that directly compares the pattern and outcomes of off-label marketing oversight by the FDA in the US with the PMCPA in the UK.

## Supporting Information

S1 Table Off-label promotion rulings by the PMCPA, 2003–2012. (DOCX)

S2 Table Off-label promotion rulings 2003–2012: Violating companies. (DOCX)

S3 Table Off-label promotion rulings 2003–2012: Drug classes. (DOCX)

## Abbreviations

ABPI: The Association of the British Pharmaceutical Industry
ATC: Anatomical Therapeutic Chemical
CD: cervical dystonia
COPD: chronic obstructive pulmonary disease
DOJ: Department of Justice
EMA: European Medicines Agency
EU: European Union
FCA: False Claims Act
FDA: Food and Drug Administration
GAO: Government Accountability Office
HA: headache
ICD: International Statistical Classification of Disease and Related Health Problems
MHRA: The Medicines and Healthcare products Regulatory Agency
MS: multiple sclerosis
PHN: postherpetic neuralgia
PMCPA: Prescription Medicines Code of Practice Authority
SPC: summary of product characteristics

## References

[1] European Union. Directive 2001/83/EC of the European Parliament and of the Council of 6 November 2001 on the Community code relating to medicinal products for human use; Article 87. http://ec.europa.eu/health/files/eudralex/vol-1/dir_2001_83_consol_2012/dir_2001_83_cons_2012_en.pdf. Accessed 28 July 2015.

[2] Radley DC, Finkelstein SN, Stafford RS. Off-label prescribing among office-based physicians. Arch Intern Med. 2006;166(9):1021–6. 10.1001/archinte.166.9.1021 16682577

[3] Ratner M, Gura T. Off-label or off-limits? Nat Biotech. 2008;26(8):867–75. 10.1038/nbt0808-867 18688236

[4] European Federation of Pharmaceutical Industries and Associations (EFPIA). Promotion of off label use of medicines by European healthcare bodies in indications where authorised medicines are available. 2014. http://www.efpia.eu/uploads/Modules/Documents/efpia-position-paper-off-label-use-may-2014.pdf. Accessed 28 July 2015.

[5] Mello MM, Studdert DM, Brennan TA. Shifting Terrain in the Regulation of Off-Label Promotion of Pharmaceuticals. New Engl J Med. 2009;360(15):1557–66. 10.1056/NEJMhle0807695 19357413

[6] Ventola CL. Off-Label Drug Information: Regulation, Distribution, Evaluation, and Related Controversies. P T. 2009;34(8):428–40. 20140107 PMC2799128

[7] Lenk C, Duttge G. Ethical and legal framework and regulation for off-label use: European perspective. Ther Clin Risk Manag. 2014;10:537–46. 10.2147/tcrm.s40232 25050064 PMC4103928

[8] Medicines & Healthcare products Regulatory Agency (MHRA). The Blue Guide: advertising and promotion of medicines in the UK; 3rd Edition. 2014. https://www.gov.uk/government/uploads/system/uploads/attachment_data/file/376398/Blue_Guide.pdf. Accessed 28 July 2015.

[9] Medicines & Healthcare products Regulatory Agency (MHRA). Memorandum of understanding between the ABPI, the PMCPA and the MHRA. 2005. http://www.abpi.org.uk/our-work/news/2011/Documents/Memorandum%20of%20Understanding%20between%20the%20ABPI,%20PMCPA%20and%20SFO%20Final%20April%202011.pdf. Accessed 27 July 2015.

[10] Zetterqvist AV, Merlo J, Mulinari S. Complaints, complainants, and rulings regarding drug promotion in the United Kingdom and Sweden 2004–2012: A quantitative and qualitative study of pharmaceutical Industry self-regulation. PLOS Med 12(2): e1001785 10.1371/journal.pmed.1001785 25689460 PMC4331559

[11] Association of the British Pharmaceutical Industry (ABPI). Code of Practice for the pharmaceutical industry. 2014. http://www.pmcpa.org.uk/thecode/Documents/PMCPA%20Code%20of%20Practice%202014.pdf. Accessed 27 July 2015.

[12] Casali PG. The off-label use of drugs in oncology: a position paper by the European Society for Medical Oncology (ESMO). Ann Oncol. 2007;18(12):1923–5. 10.1093/annonc/mdm517 18083693

[13] Levêque D. Off-label use of anticancer drugs. Lancet Oncol. 2008;9(11):1102–7. 10.1016/S1470-2045(08)70280-8. 10.1016/S1470-2045(08)70280-8 19012859

[14] Anon. Off-label prescriptions: patient safety first. Lancet Oncol. 2011;12(9):825 10.1016/S1470-2045(11)70246-7 21875558

[15] Martin-Latry K, Ricard C, Verdoux H. A one-day survey of characteristics of off-label hospital prescription of psychotropic drugs. Pharmacopsychiatry. 2007;40(03):116–20. 10. doi: 1055/s-2007-977713 17541887 10.1055/s-2007-977713

[16] Kaye JA, Bradbury BD, Jick H. Changes in antipsychotic drug prescribing by general practitioners in the United Kingdom from 1991 to 2000: a population-based observational study. Br J Clin Pharmacol. 2003;56(5):569–75. 10.1046/j.1365-2125.2003.01905.x 14651732 PMC1884397

[17] Conroy S, Choonara I, Impicciatore P, Mohn A, Arnell H, Rane A, et al Survey of unlicensed and off label drug use in paediatric wards in European countries. European Network for Drug Investigation in Children. BMJ. 2000;320(7227):79–82. 10625257 10.1136/bmj.320.7227.79 PMC27251

[18] Conroy S, Newman C, Gudka S. Unlicensed and off label drug use in acute lymphoblastic leukaemia and other malignancies in children. Ann Oncol. 2003;14(1):42–7. 10.1093/annonc/mdg031 12488291

[19] Neubert A, Wong ICK, Bonifazi A, Catapano M, Felisi M, Baiardi P, et al Defining off-label and unlicensed use of medicines for children: Results of a Delphi survey. Pharmacol Res. 2008;58(5–6):316–22. 10.1016/j.phrs.2008.09.007. 10.1016/j.phrs.2008.09.007 18852048

[20] Culshaw J, Kendall D, Wilcock A. Off-label prescribing in palliative care: A survey of independent prescribers. Palliat Med. 2013;27(4):314–9. 10.1177/0269216312465664 23175511

[21] Pavis H, Wilcock A. Prescribing of drugs for use outside their licence in palliative care: survey of specialists in the United Kingdom. BMJ. 2001;323(7311):484–5. 10.1136/bmj.323.7311.484 11532839 PMC48132

[22] Walton SM, Schumock GT, Lee KV, Alexander GC, Meltzer D, Stafford RS. Prioritizing future research on off-label prescribing: results of a quantitative evaluation. Pharmacotherapy. 2008;28(12):1443–52. 10.1592/phco.28.12.1443 19025425 PMC4406412

[23] Fugh-Berman A, Melnick D. Off-Label Promotion, On-Target Sales. PLOS Med. 2008;5(10):e210 10.1371/journal.pmed.0050210 18959472 PMC2573913

[24] Moja L, Lucenteforte E, Kwang KH, Bertele V, Campomori A, Chakravarthy U, et al Systemic safety of bevacizumab versus ranibizumab for neovascular age-related macular degeneration. Cochrane Database Syst Rev. 2014;9:CD011230 10.1002/14651858.CD011230.pub2 25220133 PMC4262120

[25] National Institute for Health and Care Excellence (NICE). Evidence summaries: unlicensed or off-label medicines. 2014. http://www.nice.org.uk/about/what-we-do/our-programmes/nice-advice/evidence-summaries-unlicensed-or-off-label-medicines Accessed 27 July 2015.

[26] Yank V, Tuohy CV, Logan AC, Bravata DM, Staudenmayer K, Eisenhut R, et al Systematic Review: benefits and harms of in-hospital use of recombinant Factor VIIa for off-label indications. Ann Intern Med. 2011;154(8):529–40. 10.7326/0003-4819-154-8-201104190-00004 21502651 PMC4102260

[27] Connolly HM, Crary JL, McGoon MD, Hensrud DD, Edwards BS, Edwards WD, et al Valvular heart disease associated with fenfluramine–phentermine. New Engl J Med. 1997;337(9):581–8. 10.1056/NEJM199708283370901 9271479

[28] Ferriman A. UK licence for cisapride suspended. BMJ. 2000;321:259. PMC1118265 10915117

[29] Matthews S. Pharma fines increase, but the pain is not felt on Wall Street. Nat Med. 2013;19(1):5 10.1038/nm0113-5 23295992

[30] Kesselheim AS, Darby D, Studdert DM, Glynn R, Levin R, Avorn J. False Claims Act prosecution did not deter off-label drug use in the case of neurontin. Health Aff. 2011;30(12):2318–27. 10.1377/hlthaff.2011.0370 22147859

[31] Larkin I, Ang D, Avorn J, Kesselheim AS. Restrictions on pharmaceutical detailing reduced off-label prescribing of antidepressants and antipsychotics in children. Health Aff. 2014;33(6):1014–23. 10.1377/hlthaff.2013.0939 24889951

[32] Steinman MA, Harper GM, Chren MM, Landefeld CS, Bero LA. Characteristics and impact of drug detailing for gabapentin. PLOS Med. 2007;4(4):743–51. 10.1371/journal.pmed.0040134 PMC1855692 17455990

[33] Lexchin J, Kohler JC. The danger of imperfect regulation: OxyContin use in the United States and Canada. Int J Risk Saf Med. 2011;23(4):233–40. 10.3233/JRS-2011-0539 22156088

[34] Kesselheim AS, Mello MM, Studdert DM. Strategies and practices in off-label marketing of pharmaceuticals: a retrospective analysis of whistleblower complaints. PLOS Med. 2011;8(4):e1000431 10.1371/journal.pmed.1000431 21483716 PMC3071370

[35] United States Government Accountability Office (GAO). FDA’s oversight of the promotion of drugs for off-label uses. 2008. http://www.gao.gov/assets/280/278832.pdf. Accessed 27 July 2015.

[36] Landefeld CS, Steinman MA. The neurontin legacy—marketing through misinformation and manipulation. New Engl J Med. 2009;360(2):103–6. 10.1056/NEJMp0808659 19129523

[37] Kesselheim AS, Studdert DM, Mello MM. Whistle-blowers’ experiences in fraud litigation against pharmaceutical companies. New Engl J Med. 2010;362(19):1832–9. 10.1056/NEJMsr0912039 20463344

[38] Almashat S, Wolfe S. Public Citizen Report: Pharmaceutical Industry Criminal and Civil Penalties: An Update. 2012. http://www.citizen.org/hrg2073 Accessed 27 July 2015.

[39] Steinman MA, Bero LA, Chren MM, Landefeld CS. Narrative review: the promotion of Gabapentin: an analysis of internal industry documents. Ann Intern Med. 2006;145(4):284–93. 10.7326/0003-4819-145-4-200608150-00008 16908919

[40] Ross JS, Hill KP, Egilman DS, Krumholz HM. Guest authorship and ghostwriting in publications related to rofecoxib: A case study of industry documents from rofecoxib litigation. JAMA. 2008;299(15):1800–12. 10.1001/jama.299.15.1800 18413874

[41] Congress of the United States. The Marketing of Vioxx to Physicians. 2005. http://democrats.oversight.house.gov/sites/democrats.oversight.house.gov/files/documents/20050505114932-41272.pdf. Accessed 27 July 2015.

[42] Prescription Medicines Code of Practice Authority (PMCPA). AUTH/2572/1/13—Ex-employee v AstraZeneca. 2013. http://www.pmcpa.org.uk/cases/Pages/2572.aspx. Accessed 27 July 2015.

[43] New Statesman. Ben Goldacre v the Association of the British Pharmaceutical Industry. New Statesman. 19 Oct 2012. http://www.newstatesman.com/sci-tech/sci-tech/2012/10/ben-goldacre-v-association-british-pharmaceutical-industry. Accessed 27 July 2015.

[44] Berelson B. Content analysis in communication research New York: Free Press; 1952.

[45] Glaser BG, Strauss AL. The Discovery of Grounded Theory: Strategies for Qualitative Research New York: Aldine de Gruyter; 1967.

[46] World Health Organization (WHO). International Statistical Classification of Disease and Related Health Problems 10th Revision, ICD-10. 2015. http://apps.who.int/classifications/icd10/browse/2015/en. Accessed 27 July 2015.

[47] Taxpayers Against Fraud Education Fund. Statistics. http://www.taf.org/resource/fca/statistics. Accessed 27 July 2015.

[48] Mulinari S. Regulating drug information in Europe: a pyrrhic victory for pharmaceutical industry critics? Sociol Health Illn. 2013;35(5):761–77. 10.1111/j.1467-9566.2012.01528.x 23094890

[49] Jack A. Letting the sunshine in on doctor-pharma relationships. BMJ. 2011;343:d6459 10.1136/bmj.d6459 21990283

[50] Herxheimer A, Collier J. Promotion by the British pharmaceutical industry 1983–8 –a critical analysis of self regulation. BMJ. 1990;300(6720):307–11. 2106963 10.1136/bmj.300.6720.307 PMC1661956

[51] Zetterqvist AV, Mulinari S. Misleading advertising for antidepressants in Sweden: a failure of pharmaceutical industry self-regulation. PLOS One. 2013;8(5). e62609 10.1371/journal.pone.0062609 23650519 PMC3641086

[52] Lexchin J. Enforcement of codes governing pharmaceutical promotion: What happens when companies breach advertising guidelines? CAMJ. 1997;156(3):351–6. PMC1226955 9033415

[53] Medicines & Healthcare products Regulatory Agency (MHRA). Advertising complaint—Pradaxa (dabigatran), promotion of unlicensed indications to healthcare professionals. April 2009.

[54] Medicines & Healthcare products Regulatory Agency (MHRA). Advertising complaint—Allergan Neurology Pharmaceutical Survey—Promotion of Botox (botulinum toxin type A) to doctors. March 2010.

[55] British Medical Journal (BMJ). BMJ media pack 2015. http://www.bmj.com/company/wp-content/uploads/2014/06/Reaching-healthcare-professionals-June-2015.pdf. Accessed 27 July 2015.

[56] Prescription Medicines Code of Practice Authority (PMCPA). Advertisements and public reprimands 2014. http://www.pmcpa.org.uk/cases/Pages/Advertisements-and-public-reprimands.aspx. Accessed 27 July 2015.

[57] Davis C, Abraham J. Is there a cure for corporate crime in the drug industry? BMJ. 2013;346:f755 10.1136/bmj.f755 23390241

[58] Gøtzsche PC. Big pharma often commits corporate crime, and this must be stopped. BMJ. 2012;345:e8462 10.1136/bmj.e8462 23241451

[59] House of Commons Health Committee (2005) The Influence of the pharmaceutical industry: Health Select Committee Report Volume 2 London: The stationery office limited 550 p. 10.1016/j.plrev.2005.01.001

[60] Chen DT, Wynia MK, Moloney RM, Alexander GC. US physician knowledge of the FDA-approved indications and evidence base for commonly prescribed drugs: results of a national survey. Pharmacoepidemiol Drug Saf. 2009;18(11):1094–100. 10.1002/pds.1825 19697444

[61] Mintzes B, Lexchin J, Sutherland J, Beaulieu MD, Wilkes M, Durrieu G, et al Pharmaceutical sales representatives and patient safety: a comparative prospective study of information quality in Canada, France and the United States. J Gen Intern Med. 2013;28(10):1368–75. 10.1007/s11606-013-2411-7 23558775 PMC3785667

[62] Avorn J, Soumerai SB. Improving drug-therapy decisions through educational outreach. A randomized controlled trial of academically based “detailing”. New Engl J Med. 1983;308(24):1457–63. 10.1056/nejm198306163082406 6406886

[63] van Eijk MEC, Avorn J, Porsius AJ, de Boer A. Reducing prescribing of highly anticholinergic antidepressants for elderly people: randomised trial of group versus individual academic detailing. BMJ. 2001;322(7287):654–7. 10.1136/bmj.322.7287.654 11250852 PMC26547

