# Supplementary material for: Pharmaceutical Industry Off-label Promotion and Self-regulation: A Document Analysis of Off-label Promotion Rulings by the United Kingdom Prescription Medicines Code of Practice Authority 2003–2012
Source: PLoS Med. 2016 Jan 26;13(1):e1001945. doi: 10.1371/journal.pmed.1001945 (PMC4727894; doi:10.1371/journal.pmed.1001945)
Supplement: S1 Table — (DOCX) [file pmed.1001945.s001.docx]

**S1 Table. Off-label promotion rulings by the PMCPA, 2003-2012**

| **Case no** | **n^1^** | **Offender** | **Complainant** | **Drug** |
| --- | --- | --- | --- | --- |
| **1440/3/03** | 1 | Pfizer | AstraZeneca | Lipitor (atorvastatin) |
| **1442/3/03** | 1 | Pfizer | Lilly | Viagra (sildenafil) |
| **1467/5/03** | 1 | Novartis | Media/  Director | Elidel (pimecrolimus) |
| **1468/5/03 & 1469/5/03** | 1 | Procter & Gamble and Aventis Pharma | Merck Sharp & Dohme | Actonel (risedronate) |
| **1506/8/03** | 1 | Lilly | Pfizer | Cialis (tadalafil) |
| **1514/9/03** | 1 | GlaxoSmithKline | Aventis Pasteur MSD | Havrix (hepatitis A virus, inactivated antigen) |
| **1477/6/03** | 3 | Novartis | Bristol-Myers Squibb & Sanofi-Synthelabo | Diovan (valsartan) |
| **1491/7/03** | 1 | Fujisawa | Novartis | Protopic (tacromilus) |
| **1526/10/03** | 3 | Roche | Novartis | Bondronat (ibandronate) |
| **1536/11/03** | 2 | Abbott Laboratories | Wyeth | Humira (adalimumab) |
| **1525/10/03** | 1 | Aventis Pharma | Pierre Fabre | Taxotere (docetaxel) |
| **1527/10/03** | 1 | Astra Zeneca | GlaxoSmithKline | Symbicort (budesonide/ formoterol fumarate dihydrate) |
| **1533/10/03** | 1 | Takeda | Servier | Actos (pioglitazone) |
| **1551/2/04** | 1 | GlaxoSmithKline | Pfizer & Boehringer Ingelheim | Seretide (salmeterol/fluticasone) |
| **1554/2/04** | 1 | Amgen | Hospital Pharmacist | Aranesp (darbepoetin alfa) |
| **1555/2/04** | 1 | Lundbeck | Primary Care Trust | Ebixa (memantine) |
| **1544/1/04** | 1 | Napp | Janssen-Cilag | OxyContin (Oxycodone) |
| **1580/4/04** | 1 | GlaxoSmithKline | Head of Primary Care Trust Prescribing Support Unit | Avandia (rosiglitazone);  Avandamet (rosiglitazone/metformin) |
| **1599/6/04** | 1 | Roche | Novartis | Bondronat (ibandronate) |
| **1613/8/04** | 1 | Johnson & Johnson Wound Management | Baxter Healthcare | Quixil (human surgical sealant) |
| **1614/8/04** | 1 | Merck Sharp & Dohme | Scrutiny/Director | Arcoxia (etoricoxib) |
| **1557/2/04** | 1 | Aventis Pharma | Pierre Fabre/Director | Taxotere (docetaxel) |
| **1623/8/04 & 1624/8/04** | 3 | Bristol-Myers Squibb & Otsuka | Lilly | Abilify (aripiprazole) |
| **1667/12/04** | 1 | Merck Sharp & Dohme | Gilead Sciences | Cancidas (caspofungin) |
| **1693/3/05 & 1694/3/05** | 1 | Lilly & Boehringer Ingleheim | Lundbeck | Cymbalta (duloxetine) |
| **1715/6/05** | 1 | Cephalon | Hospital Consultant | Actiq (fentanyl citrate) |
| **1736/7/05** | 1 | Abbott | Roche | Reductil (sibutramine) |
| **1754/9/05** | 1 | Merck | Anonymous Medical Representative | Niaspan (niacin) |
| **1708/5/05** | 1 | Serano | Brogen Idec | Rebif (interferon beta-1a) |
| **1777/10/05** | 1 | Schering Health Care | Anonymous | Betaferon (interferon beta-1b) |
| **1801/2/06** | 1 | GlaxoSmithKline | General practitioner | Requip (ropinirole) |
| **1813/3/06** | 3 | Bayer | Lilly | Levitra (vardenafil) |
| **1851/6/06** | 1 | Novartis | Roche | Myfortic (mycophenolate sodium) |
| **1871/7/06** | 1 | Sanofi-Aventis | Doctor | Acomplia (rimonabant) |
| **1960/2/07** | 1 | Grünenthal | Consultant in Anaesthesia and Pain Management | Versatis (lidocaine) |
| **2008/6/07** | 2 | ProStrakan | Member of the public/Director | Rectogesic (glyceryl trinitrate) |
| **2059/10/07** | 1 | Janssen –Cilag | Primary care Trust Medicines Management Director | Risperdal Consta (risperidone) |
| **2102/3/08** | 1 | Meda | Anonymous representative | Aldara (imiquimod) |
| **2119/4/08** | 1 | Merz Pharma | Allergan | Xeomin (clostridium botulinum type A neurotoxin) |
| **2125/5/08** | 1 | Takeda Europe | GlaxoSmithKline | Actos (pioglitazone) |
| **2156/8/08** | 1 | Sanofi Pasteur MSD | GlaxoSmithKline | Gardsasil (capsid protein L1, human papillomavirus (HPV), type 6, 11, 16, 18) |
| **2185/11/08** | 1 | Schering-Plough | Pharmacist-Practitioner | NeoClarityn (desloratadine) |
| **2164/9/08** | 1 | Takeda | Merck Sharp & Dohme | Actos (pioglitazone)  Competact (pioglitazone and metformin) |
| **2206/2/09** | 1 | Voluntary admission by AstraZeneca | | Crestor (rosuvastatin) |
| **2211/3/09** | 1 | Boehringer Ingelheim | General practitioner | Micardis (telmisartan) and Micardis Plus (telmisartan and hydrochlorothiazide) |
| **2209/2/09** | 1 | Leo Pharma | Pfizer | Innohep (tinzaparin) |
| **2215/3/09** | 2 | Allergan | Merz Pharma | Botox (botulinum neurotoxin) |
| **2218/3/09** | 2 | Voluntary Admission by AstraZeneka | | Nexium (esomeprazole) |
| **2231/5/09** | 3 | Boehringe Ingelheim | Bayer | Pradaxa (dabigatran) |
| **2244/6/09** | 1 | Stiefel | General practitioner & pharmacist | Duac (clindamycin and benzoyl peroxide) |
| **2246/7/09** | 1 | Novartis | Roche/Director | Zometa (zoledronic acid) |
| **2263/9/09** | 1 | Boehringer Ingelheim | Bristol-Myers Squibb | Viramune (nevirapine) |
| **2267/9/09** | 2 | Procter & Gamble | Shire | Asacol (mesalazine) |
| **2274/10/09** | 1 | Allergan | Consultant Neurologist | Botox (botulinum neurotoxin) |
| **2273/10/09** | 2 | Novo Nordisk | Lilly | Victoza (liraglutide) |
| **2293/1/10** | 1 | Voluntary Admission by Ferring | | Pentasa (mesalazine) |
| **2299/2/10** | 1 | Ferring | Shire | Pentasa (mesalazine) |
| **2313/4/10** | 1 | Chiesi | Teva | Clenil (beclometasone dipropionate) |
| **2312/4/10** | 1 | Pfizer | Allergan | Xalatan (latanoprost) and Xalacom (latanoprost plus timomol) |
| **2330/7/10** | 1 | Grünenthal | Anonymous | Versatis (lidocaine) |
| **2331/7/10** | 1 | Lilly | Novo Nordisk | Byetta (exenatide) |
| **2334/7/10** | 1 | Movetis | Norgine | Resolor (prucalopride) |
| **2338/7/10** | 1 | Dexcel Pharma | ESPRIT | Deximune (ciclosporin) |
| **2340/7/10** | 2 | Dexcel Pharma | Novartis | Deximune (ciclosporin) |
| **2379/1/11** | 1 | Chiesi | Anonymous | Fostair (beclometasone and formoterol) |
| **2383/2/11** | 1 | Bayer | Anonymous General Practitioner | Yasmin (ethinylestradiol and drospirenone) |
| **2394/3/11 & 2395/3/11** | 1 | Lundbeck & Teva | Boehringer Ingelheim | Azilect (rasagiline) |
| **2404/5/11** | 1 | Boehringer Ingelheim | General Practitioner | Pradaxa (dabigatran) |
| **2417/6/11** | 1 | Servier | Primary Care Trust Head of Medicines Management | Procoralan (ivabradine) |
| **2469/12/11** | 1 | Voluntary admission by Bayer | | Levitra (vardenafil) |
| **2483/2/12** | 1 | Pfizer | Consultant in sexual health | Prevenar 13 (Pneumococcal polysaccharide conjugate vaccine (13-valent, adsorbed) |
| **2474/1/12** | 1 | GlaxoSmithKline | GlaxoSmithKline employee | Revolade (eltrombopag)  Seretide (fluticasone/salmeterol)  reQuip XL (ropinirole) |
| **2506/5/12 & 2507/5/12** | 4 | Lilly & Daiichi-Sankyo | AstraZeneca | Efient (prasugrel) |
| **2435/8/11** | 2 | Chiesi | GlaxoSmithKline /Director | Fostair (beclometasone and formoterol) |

^1^ Matters in breach per case
