# Supplementary material for: Pharmaceutical Industry Off-label Promotion and Self-regulation: A Document Analysis of Off-label Promotion Rulings by the United Kingdom Prescription Medicines Code of Practice Authority 2003–2012
Source: PLoS Med. 2016 Jan 26;13(1):e1001945. doi: 10.1371/journal.pmed.1001945 (PMC4727894; doi:10.1371/journal.pmed.1001945)
Supplement: S2 Table — (DOCX) [file pmed.1001945.s002.docx]

**Table S2. Off-label promotion rulings, 2003-2012: violating pharmaceutical companies**

| **Company** | **Cases/Matters** |
| --- | --- |
| Boehringer Ingelheim | 5(7) |
| GlaxoSmithKline | 5(5) |
| Lilly | 4(7) |
| Novartis | 4(6) |
| Pfizer | 4(4) |
| Bayer | 3(5) |
| AstraZeneca | 3(4) |
| Chiesi | 3(4) |
| Aventis Pharma (Sanofi Aventis Group) | 3(3) |
| Takeda | 3(3) |
| Roche | 2(4) |
| Procter & Gamble | 2(3) |
| Dexcel Pharma | 2(3) |
| Allergan | 2(3) |
| Abbott | 2(3) |
| Merck Sharp & Dohme | 2(2) |
| Grünenthal | 2(2) |
| Lundbeck | 2(2) |
| Ferring | 2(2) |
| Daiichi-Sankyo | 1(4) |
| Bristol-Myers Squibb | 1(3) |
| Otsuka | 1(3) |
| Novo Nordisk | 1(2) |
| ProStrakan | 1(2) |
| Johnson & Johnson Wound Management | 1(1) |
| Janssen-Cilag (Johnson & Johnson subsidiary) | 1(1) |
| Teva | 1(1) |
| Fujisawa | 1(1) |
| Merck | 1(1) |
| Amgen | 1(1) |
| Napp | 1(1) |
| Merz Pharma | 1(1) |
| Leo Pharma | 1(1) |
| Servier | 1(1) |
| Meda | 1(1) |
| Cephalon | 1(1) |
| Serano | 1(1) |
| Schering Health Care | 1(1) |
| Sanofi-Aventis (Sanofi-Aventis Group) | 1(1) |
| Sanofi/Sanofi Pasteur MSD (Sanofi-Aventis Group) | 1(1) |
| Schering-Plough | 1(1) |
| Stiefel (GlaxoSmithKline subsidiary) | 1(1) |
| Movetis | 1(1) |
