# Supplementary material for: Pharmaceutical Industry Off-label Promotion and Self-regulation: A Document Analysis of Off-label Promotion Rulings by the United Kingdom Prescription Medicines Code of Practice Authority 2003–2012
Source: PLoS Med. 2016 Jan 26;13(1):e1001945. doi: 10.1371/journal.pmed.1001945 (PMC4727894; doi:10.1371/journal.pmed.1001945)
Supplement: S3 Table — (DOCX) [file pmed.1001945.s003.docx]

**S3 Table. Off-label promotion rulings 2003-2012: drug classes**

| **Therapeutic drug class/ATC** | **Cases/Matters** | **Drug(s)** |
| --- | --- | --- |
| Diabetes drugs (A10) | 7(8) | Actos (pioglitazone); Avandia (rosiglitazone); Avandamet (rosiglitazone/metformin); Competact (pioglitazone and metformin); Byetta (exenatide) |
| Drugs for obstructive airway diseases (R03) | 5(6) | Seretide (fluticasone/salmeterol); Fostair (beclometasone and formoterol); Symbicort (budesonide/formoterol fumarate dihydrate) |
| Antithrombotic agents (B01) | 4(9) | Innohep (tinzaparin); Pradaxa (dabigatran); Efient (prasugrel) |
| Drugs for treatment of bone diseases (M05) | 4(6) | Actonel (risedronate); Bondronat (ibandronate); Zometa (zoledronic acid) |
| Immunosuppressants (L04) | 4(6) | Humira (adalimumab); Myfortic (mycophenolate sodium); Deximune (ciclosporin) |
| Urological drugs (G04) | 4(6) | Levitra (vardenafil); Viagra (sildenafil); Cialis (tadalafil) |
| Antidiarrheals, intestinal anti-inflammatory/anti-infective agents (A07) | 3(4) | Pentasa (mesalazine); Asacol (mesalazine) |
| Muscle relaxants (M03) | 3(4) | Xeomin (clostridium botulinum type A neurotoxin); Botox (botulinum neurotoxin) |
| Lipid modifying agents (C10) | 3(3) | Lipitor (atorvastatin); Niaspan (niacin); Crestor (rosuvastatin) |
| Anti-Parkinson drugs (N04) | 3(3) | reQuip XL (ropinirole); Azilect (rasagiline); Requip XL (ropinirole) |
| Vaccines (J07) | 3(3) | Elidel (hepatitis A virus, inactivated antigen); Gardasil (capsid protein L 1, human papillomavirus (HPV), type 6, 11, 16, 18);  Prevenar 13 (Pneumococal polysaccharide conjugate vaccine (13-valent, absorbed) |
| Psycholeptics drugs (N05) | 2(4) | Abilify (aripiprazole); Risperdal Consta (risperidone) |
| Agents acting on the renin-angiotensin system (C09) | 2(4) | Micardis (telmisartan) and Micardis Plus (telmisartan and hydrochlorothiazide); Diovan (valsartan) |
| Antineoplastic agents (L01) | 2(2) | Taxotere (docetaxel) |
| Anesthetics (N01) | 2(2) | Versatis (lidocaine) |
| Immunostimulants (L03) | 2(2) | Rebif (interferon beta-1a); Betaferon (interferon beta-1b) |
| Antihemorrhagics (B02) | 2(2) | Revolade (eltrombopag); Quixil (human surgical sealant) |
| Other dermatological preparations (D11) | 2(2) | Elidel (pimecrolimus); Protopic (tacromilus) |
| Psychoanaleptics (N06) | 2(2) | Ebixa (memantine); Cymbalta (duloexetine) |
| Analgesics (N02) | 2(2) | OxyContin (Oxycodone); Actiq (fentanyl citrate) |
| Antiobesity preparations (A08) | 2(2) | Reductil (sibutramine); Acomplia (rimonabant) |
| Vasoprotectives (C05) | 1(2) | Rectogesic (glyceryl trinitrate) |
| Drugs for acid related disorders (A02) | 1(2) | Nexium (esomeprazole) |
| Blood substitutes and perfusion solutions (B05) | 1(1) | Clenil (beclometasone diproppionate) |
| Sex hormones and modulators of the genital system (G03) | 1(1) | Yasmin (ethinylestradio 1 and drospirenone) |
| Antimycotics for systemic use (J02) | 1(1) | Cancidas (caspofungin) |
| Drugs for constipation (A06) | 1(1) | Resolor (prucalopride) |
| Ophthalmologicals (S01) | 1(1) | Xalatan (latanoprost) and Xalacom (latanoprost plus timomol) |
| Cardiac therapy (C01) | 1(1) | Procooralan (ivabradine) |
| Antibiotics and chemotherapeutics for dermatological use (D06) | 1(1) | Aldara (imiquimod) |
| Antianemic preparations (B03) | 1(1) | Aranesp (darbepoetin) |
| Antihistamines for systemic use (R06) | 1(1) | NeoClarityn (desloratadine) |
| Anti-acne preparations (D10) | 1(1) | Duac (clindamycin and benzoyl peroxide) |
| Antivirals for systemic use (J05) | 1(1) | Viramune (nevirapine) |
| Anti-inflammatory and antirheumatic (M01) | 1(1) | Arcoxia (etoricoxib) |
